# Urban–rural inequalities in suicide mortality: a comparison of urbanicity indicators

**DOI:** 10.1186/s12942-017-0112-x

**Published:** 2017-10-30

**Authors:** M. Helbich, V. Blüml, T. de Jong, P. L. Plener, M.-P. Kwan, N. D. Kapusta

**Affiliations:** 1 Department of Human Geography and Spatial Planning, Utrecht University, Heidelberglaan 2, 3584 CS Utrecht, The Netherlands; 2 Department of Psychoanalysis and Psychotherapy, Medical University of Vienna, 1090 Vienna, Austria; 3 Department of Child and Adolescent Psychiatry and Psychotherapy, University of Ulm, 89075 Ulm, Germany; 4 Department of Geography and Geographic Information Science, University of Illinois at Urbana–Champaign, Urbana, IL 61801 USA; 5 Department of Logistics, University of Stellenbosch, Van der Sterrbuilding 3017, Bosmanstreet, Matieland, Stellenbosch, South Africa

**Keywords:** Suicide mortality, Spatial inequalities, Urban–rural differences, Indicator comparison, Germany

## Abstract

**Background:**

Urban–rural disparities in suicide mortality have received considerable attention. Varying conceptualizations of urbanity may contribute to the conflicting findings. This ecological study on Germany assessed how and to what extent urban–rural suicide associations are affected by 14 different urban–rural indicators.

**Methods:**

Indicators were based on continuous or *k*-means classified population data, land-use data, planning typologies, or represented population-based accessibility indicators. Agreements between indicators were tested with correlation analyses. Spatial Bayesian Poisson regressions were estimated to examine urban–rural suicide associations while adjusting for risk and protective factors.

**Results:**

Urban–rural differences in suicide rates per 100,000 persons were found irrespective of the indicator. Strong and significant correlation was observed between different urban–rural indicators. Although the effect sign consistently referred to a reduced risk in urban areas, statistical significance was not universally confirmed by all regressions. Goodness-of-fit statistics suggested that the population potential score performs best, and that population density is the second best indicator of urbanicity. Numerical indicators are favored over classified ones. Regional planning typologies are not supported.

**Conclusions:**

The strength of suicide urban–rural associations varies with respect to the applied indicator of urbanicity. Future studies that put urban–rural inequalities central are recommended to apply either unclassified population potentials or population density indicators, but sensitivity analyses are advised.

**Electronic supplementary material:**

The online version of this article (10.1186/s12942-017-0112-x) contains supplementary material, which is available to authorized users.

## Background

Reducing mental health disparities between urban and rural settings is receiving considerable attention in both scientific and policy debates [1–4], as is suicide mortality [5–9]. In industrialized nations, suicide is a major cause of death [10], whereas suicide rates vary greatly across regions [11–14].

Various factors explain geographic variations in suicide prevalence. Meta-analyses suggest that suicidal behavior is affected by, but not limited to, socio-demographics, access to health services, and the presence of psychiatric disorders [15–17]. Whereas the living environment also seems to have neurobiological effects that contribute to differences in psychiatric illness [18], research has also found that urbanity/rurality shapes intra-regional differences in suicide [12, 14, 19–26]. There are many possible explanations for an increased suicide risk in rural areas [9, 12, 14, 20, 26, 27]. For instance, despite popular clichés about anonymous city-dwelling, rural living can lead to social isolation, resulting in less intimate face-to-face contact with family and friends, which, in turn, increases the risk for suicidal behavior [19]. Rural dwellers have easier access to lethal means, which increases their suicide risk [5]. Country living is often related to a lower socioeconomic status as well as stigmatized attitudes toward visiting mental healthcare facilities (e.g., general practitioner (GP), psychiatrists), and long travel distances diminish the demands for specialized healthcare providers [14, 28]. Several empirical studies emphasized an elevated vulnerability in rural areas [9, 12, 14, 26, 27], whereas others drew an opposite conclusion [21, 23, 29].

There are at least two reasons for these inconsistent findings about urban–rural inequalities in suicide mortality. First, urbanicity and rurality are multifaceted concepts: Neither has a universally accepted definition [30–34]. Urbanicity/rurality is frequently represented through population density, either considered as a continuous variable [13] or converted into an ordinal scaled variable using arbitrary cut-off points or the distribution of the data (e.g., natural breaks) [23, 25]. Several alternatives are available to demarcate territorial space [30–32, 34]. Planning-based typologies [22] categorize municipal jurisdictions into urban, suburban, and rural areas by means of density threshold values, morphometric descriptions, etc. Such approaches fail to represent the spatial interaction between territorial units. Accessibility-oriented urban–rural indicators such as the population potential score [35, 36], which refers to how many people can be reached within certain travel times, have received virtually no attention in suicide epidemiology [21].

Second, there is no consensus on which data sources should be used to define urban–rural areas. National statistical offices often facilitate an ad hoc application of population-based density indicators [7, 14, 37, 38]. Less readily available, but equally valid for urban–rural demarcations, are urban form features (e.g., the amount of built-up area). Although this information can be extracted from the cadaster, advances in satellite imagery have resulted in datasets describing land-use at high levels of spatial resolution [39, 40]. However, although scientifically exact methodologies for data compilation are followed, the derived urban–rural indicators differ in granularity and scale, the minimum mapping units, and the level of generalization [41], which translates to different urban–rural indicators.

Taken together, studies addressing urban–rural differences in suicide have mostly been restricted to a single indicator. None of them, to our knowledge, considered the consequences of choosing one urban–rural definition or another. Thus, it remains unclear whether and, if so, how different ways of operationalizing urbanicity affect urban–rural suicide associations. Inappropriate urban–rural indicators may potentially obscure or modify “true” urban–rural suicide associations [3, 22] and bias conclusions, leading to inappropriate health policies [32]. Our research questions were as follows:

1. To what extent do 14 different urban–rural indicators derived from different data sources correlate?
2. Do suicide mortality rates vary across different urban–rural typologies?
3. Do the nature and the strength of urban–rural suicide associations differ across indicators?

In order to address these pressing questions, we conducted an ecological study on Germany for the period 2007–11. The rationale for selecting Germany is twofold. First, Germany experienced an increase in suicides in 2007–11 [9, 42], despite the country’s suicide prevention program [43]. Second, whereas several Anglophone studies [8, 19, 21, 24, 44, 45] and Asian studies [6, 7, 27, 37] exist, research on intra-national differences in continental Europe is underrepresented [14, 38, 46].

## Methods

### Study design and data

This study was based on a cross-sectional study design at a district level for Germany (*N* = 402). These territorial units permit detailed analyses while respecting data protection laws. For each district, suicide mortality data for the period 2007–11 were obtained from the Statistical State Office of the Free State of Saxony. Following the International Classification of Diseases (10th revision), suicide cases were defined as incidents of intentional self-harm leading to death (i.e., X60–X84). The dataset comprised all suicides of persons residing in Germany who were issued a death certificate by an authorized physician [47]. As suicide data per district are sparse, and to circumvent stochastic annual variations, the average annual number of suicide cases per district was determined [11]. A similar procedure was employed for the population at risk (2007–11; German Federal Statistical Office) to determine the expected number of suicides (i.e., multiplying the German-wide suicide rate for the observation period by the average population size of each district).

The first urban–rural indicator reflects the population density (i.e., people per district; German Federal Statistical Office) for 2011. Second, we developed an indicator describing the proportion of built-up areas (e.g., residential, commercial, and industrial buildings) and transportation areas (e.g., roads, railroads, airports) per district for 2011 (in %). Input data stem from the ATKIS digital landscape model, which is accessible through the Leibniz Institute of Ecological Urban and Regional Development (IOER). Third, the proportion of built-up area per district (in %) in 2012 was computed using the Corine land-use inventory.^1^ This repository is hosted by the European Environmental Agency. Fourth, a regional typology published by the Federal Institute for Research on Building, Urban Affairs and Spatial Development (BBSR) (2011) was considered. The indicator is based on the structural characteristics of settlement areas and is composed of multiple features (e.g., population, population densities, and the proportion of people in large and mid-sized cities). This typology comprises four areas: rural areas, rural areas with densification, urbanized areas, and urban areas (i.e., major cities). We also reduced this typology to three (i.e., urban, rural areas with densification, and rural areas) and two clusters (i.e., urban and rural areas). Fifth, two accessibility indicators were implemented [36, 48]. The cumulative population opportunity index^2^ represents the number of people reachable within a 60-min car drive. The higher the opportunity index, the better the accessibility. The population potential score,^3^ in contrast, assumes a stronger influence of the nearby population compared with a population that is farther away [35]. Impedance is measured through a negative power function with a moderate distance decay of power two and automobile-based travel times [36]. The higher the potential score, the higher the population concentration. Both accessibility indicators are based on ESRI’s street network dataset 2008. Finally, we considered a European-wide urban–rural typology [49] grounding on the Geostat population grid derived through dasymetric modeling for the year 2011 [50]. This typology comprises predominantly rural areas, intermediate areas, and predominantly urban areas.

**Fig. 1:**
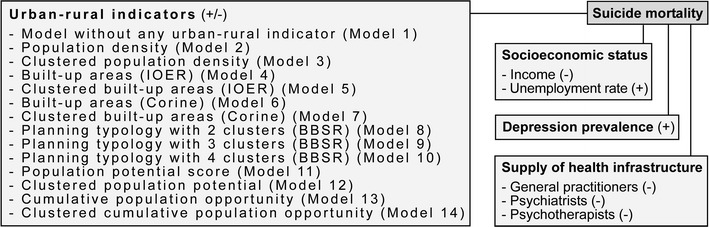
Conceptual model (A “+” refers to a positive association and a “−” refers to a negative association)

The following covariates per district were considered [17]. Data on the average disposable annual income per person (in €1000) [37] and the unemployment rate (in %) [38] for the year 2011 were acquired from the German Federal Statistical Office. Depression prevalence (in %) for 2011 was obtained from the Central Research Institute of Ambulatory Health Care [11]. Finally, data representing the supply of health infrastructure (i.e., number of general practitioners, psychiatrists, and psychotherapists per 100,000 persons) [51] for 2011 were acquired from the German Central Research Institute of Ambulatory Health Care. Figure 1 summarizes the underlying conceptual model.

### Statistical analysis

#### Urban–rural classification

To avoid arbitrary class breaks [31], the continuous urban–rural indicators were further classified by *k*-means clustering [52]. Districts were assigned to mutually exclusive regions through maximizing the internal similarity of each cluster (i.e., region). To determine an appropriate number of regions, Bayesian hierarchical models (see below) conditioned on the covariates were estimated with two to 19 regions. For each model, goodness-of-fit criteria (i.e., the deviance information criterion (DIC) [53]) and the predictive performance (i.e., the conditional predictive ordinate (CPO) [54]) were determined. Lower DIC scores refer to a better fit. Higher CPO scores indicate better predictive performance. The best model is assumed to have the most suitable number of regions.

#### Descriptive and bivariate analyses

Suicide rates per 100,000 people were cross-compared between urban and rural typologies. To quantify the relationships between the urban–rural indicators, Spearman rank correlation coefficients were computed. Correlations with *p* < 0.01 were considered statistically significant.

#### Multivariate regressions

To test the associations between suicide and individual urban–rural indicators, ecological Bayesian regressions were implemented [55, 56]. For suicide counts, the Poisson distribution is well suited and the expected number of suicides served as offset. Studies [38, 45, 55] have demonstrated that suicide risk explanation by covariates is improved by including spatial effects that otherwise bias model output. Thus, the models also comprised a spatially structured and a spatially unstructured district-specific effect while adjusting for other risk and protective factors [57]. Districts were considered neighbors if they shared a common boundary [58]. Relative risk estimates were obtained by exponentiating the posterior means together with the 95% credibility intervals (CI). A relative risk was considered significant if the 95% CI did not include one. The district-specific smoothed residual relative risk was obtained by exponentiating the sum of the structured and the unstructured spatial effect. The uncertainty related to the posterior means of the district-specific effect was also visualized [59]. Model quality was addressed with DIC and CPO scores. The models were estimated with integrated nested Laplace approximation [54, 60]. Statistical analyses were carried out using the R-INLA library (17.06.20) in R-3.3.1.

A model without any urban–rural indicator (Model 1) and 14 adjusted models with different urban–rural indicators were tested. Model 2–3 (“Census”) used the continuous and the clustered population density, model 4–5 (“IOER”) used continuous and clustered built-up and transportation areas, model 6–7 (“Corine”) used continuous and clustered Corine-based build-up areas, model 8–10 (“BBSR”) used planning typologies, model 11–12 (“Potential”) used continuous and clustered population potential scores, model 13–14 (“Opportunity”) used the continuous and clustered cumulative opportunity index, and model 15 (“ESTAT”) used the European-wide urban–rural typology. Continuous urban–rural indicators were log-transformed to correct for the skewness [19].

## Results

### Urban–rural indicators

**Fig. 2:**
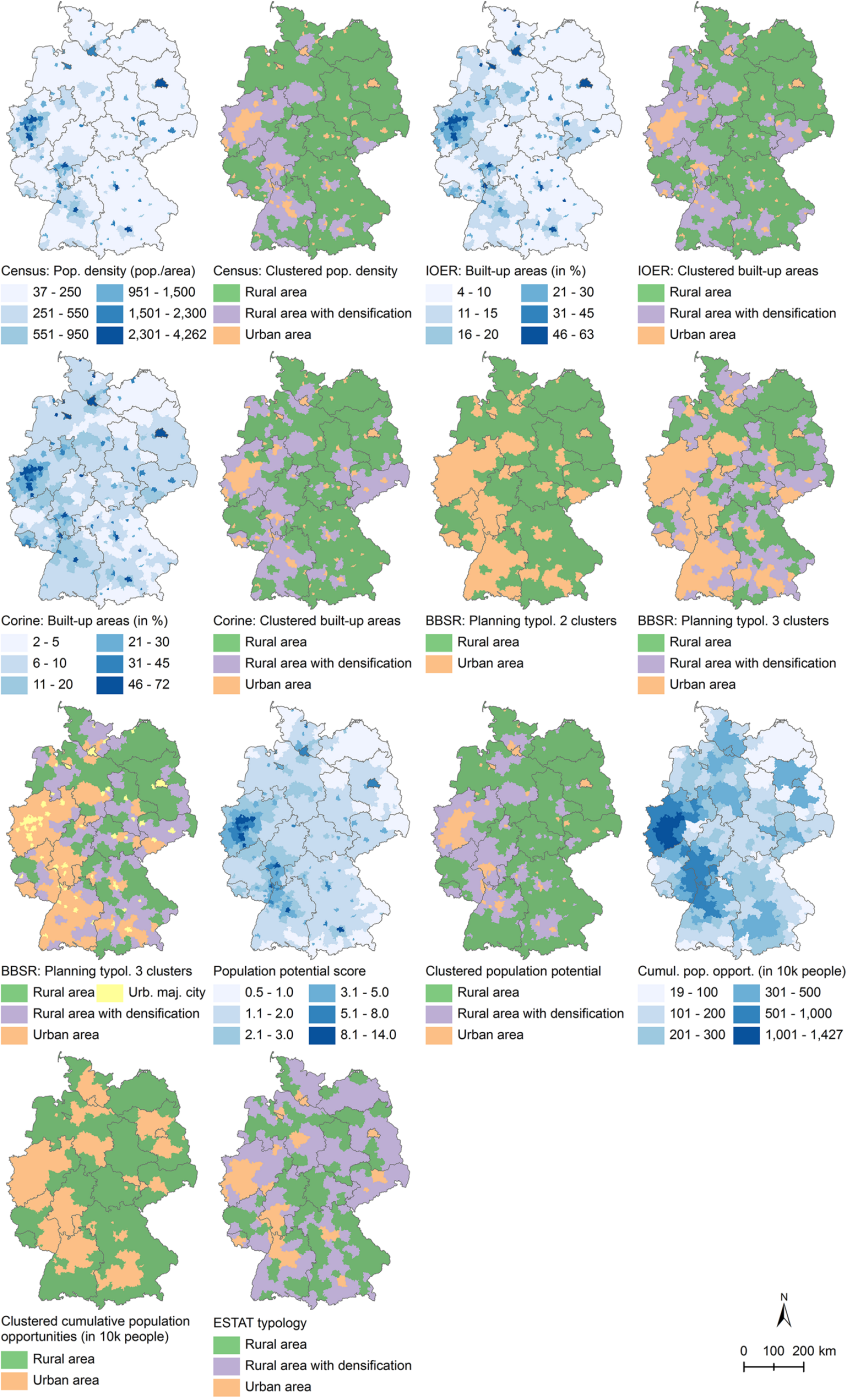
Urban–rural indicators

The five continuous urban–rural indicators were clustered and resulted in 14 operationalizations. With the exception of the opportunity indicator, both DIC and CPO values indicate that three clusters (i.e., regions) are appropriate (Additional file 1: Figure A1). Across the models, the potential score is competitive. Figure 2 visualizes the urban–rural indicators. Further descriptive statistics are provided in the Additional file 1: Table A1.

Spearman correlations (Additional file 1: Table A2) supported the visual agreements between the urban–rural indicators. The highest correlations (of > 0.9 (*p* < 0.001)) are between the continuous variables, whereas the planning-based urban–rural measures (BBSR) are less, but still highly significantly (*p* < 0.001), correlated (Table 1).

**Table 1:** Suicide rates per 100,000 people for urban–rural typologies

	#3: Census (pop. density clustered in 3 regions)	#5: IOER (built-up areas clustered in 3 regions)	#7: Corine (built-up areas clustered in 3 regions)	#8: BBSR (typol. with 2 regions)	#9: BBSR (typol. with 3 regions)	#10: BBSR (typol. with 4 regions)	#12: Pop. potential scores (clustered in 3 regions)	#14: Cumulative opport. index (clustered in 2 regions)	#15: ESTAT (typol. with 3 regions)
Rural areas	12.9	12.9	12.6	12.8	12.9	12.9	13.0	13.0	13.0
Rural areas with densification	11.6	11.8	12.0	–	12.8	12.8	11.6	–	12.2
Urban areas	11.3	11.2	11.3	11.5	11.6	11.5	11.0	11.2	11.2
Major cities	–	–	–	–	–	11.5	–	–	

Suicide rates are further stratified by different urban–rural typologies. Rural areas have higher suicide rates, namely of between 12.6 and 13.2 per 100,000 persons, compared to urban areas, where suicide rates range from 11.0 to 11.6 per 100,000 persons (Table 2). Minor fluctuations appear across the urban–rural indicators. To get more reliable insights beyond descriptive comparisons, the effects of the 14 urban–rural indicators on suicide risk were tested in multivariate models.

**Table 2:** Regression results for urban–rural indicators

Model	Urban–rural indicator		Relative risk	2.5% CI	97.5% CI
#2	Census (logged pop. density)	Continuous variable	0.943*	0.909	0.980
#3	Census (pop. density clustered in 3 regions) (Ref. rural area)	Rural areas with densification	0.940	0.882	1.000
		Urban areas	0.853*	0.775	0.940
#4	IOER (logged built-up areas %)	Continuous variable	0.919*	0.864	0.979
#5	IOER (built-up areas clustered in 3 regions) (Ref. rural area)	Rural areas with densification	0.958	0.902	1.017
		Urban areas	0.854*	0.771	0.946
#6	Corine (logged built-up areas %)	Continuous variable	0.944*	0.896	0.995
#7	Corine (built-up areas clustered in 3 regions) (Ref. rural area)	Rural areas with densification	0.979	0.921	1.040
		Urban areas	0.887*	0.804	0.978
#8	BBSR (typol. with 2 regions) (Ref. rural area)	Urban areas	0.954	0.896	1.016
#9	BBSR (typol. with 3 regions) (Ref. rural area)	Rural areas with densification	0.978	0.908	1.053
		Urban areas	0.940	0.869	1.017
#10	BBSR (typol. with 4 regions) (Ref. rural area)	Rural areas with densification	0.978	0.908	1.053
		Urbanized areas	0.946	0.875	1.024
		Urban area (major city)	0.883*	0.787	0.990
#11	Pop. potential scores (logged)	Continuous variable	0.903*	0.854	0.955
#12	Pop. potential scores (clustered in 3 regions) (Ref. rural area)	Rural areas with densification	0.922*	0.867	0.981
		Urban areas	0.850*	0.774	0.934
#13	Cumulative opport. index (logged)	Continuous variable	0.940*	0.902	0.981
#14	Cumulative opport. index (clustered in 2 regions) (Ref. rural area)	Urban areas	0.928*	0.876	0.983
#15	ESTAT (typol. with 3 regions) (Ref. rural area)	Rural areas with densification	0.973	0.913	1.036
		Urban areas	0.943	0.865	1.028

The models are adjusted for risk and protective factors. A relative risk labeled as “*” refer to a significant association. Model #1 does not adjust for urban–rural differences

### Multivariate regressions

**Fig. 3:**
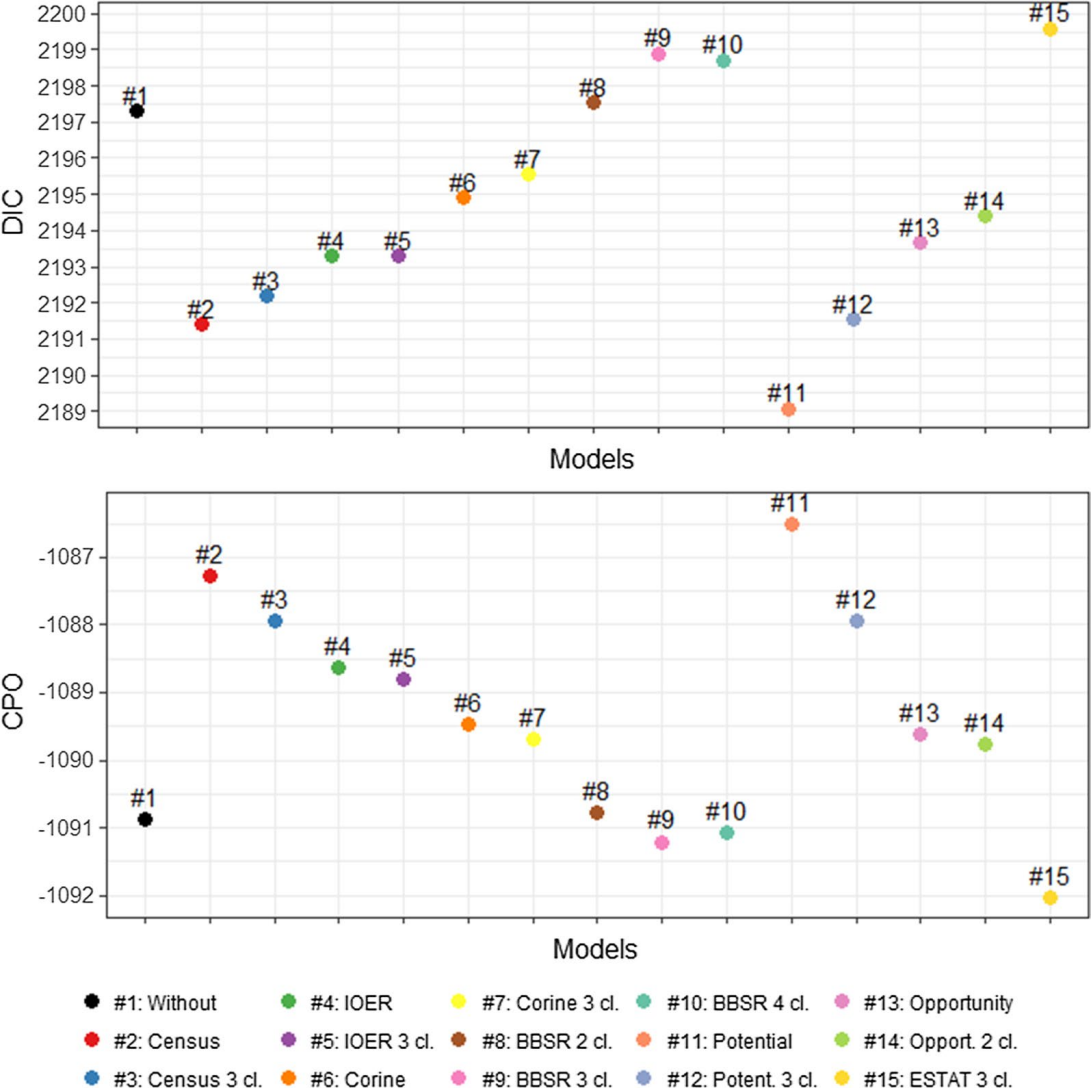
Model fits and predictive performance

Model performances of the regressions are reported in Fig. 3. Model 11 (i.e., numeric population potential score) has the highest goodness-of-fit and models 8–10 and 15 have the poorest fit (i.e., planning typologies). With DIC score differences of 8.5, statistical support is evident. Less clear is the DIC difference between model 2 and model 11 and between models 2–3 and model 4–5. The CPO values confirm these results.

Table 2 summarizes the regression results for each urban–rural specification. All models with continuous urban–rural indicators (i.e., models 2, 4, 6, 11, 13) indicate strong statistical evidence of negative associations. The magnitudes of the coefficients are roughly comparable, whereas model 11 shows the strongest negative effect (0.903, 95% CI 0.854–0.955). For the categorical urban–rural indicators, the results are less distinct. Models 3, 5, 7, and 10 support that rural areas are at higher risk than urban ones, but rural areas with densification largely remain “insignificant.” The urban–rural effects of these models range from 0.853 (95% CI 0.775–0.940) to 0.887 (95% CI 0.804–0.978), with a tendency to be lower than the effects obtained through a continuous indicator. No support for urban–rural differences in suicide rates is provided by models 8, 9, and 15, which represent planning-based typologies. Only the four-areas typology (Model 10) reveals differences between major cities and rural areas (0.883; 95% CI 0.787–0.990).

**Fig. 4:**
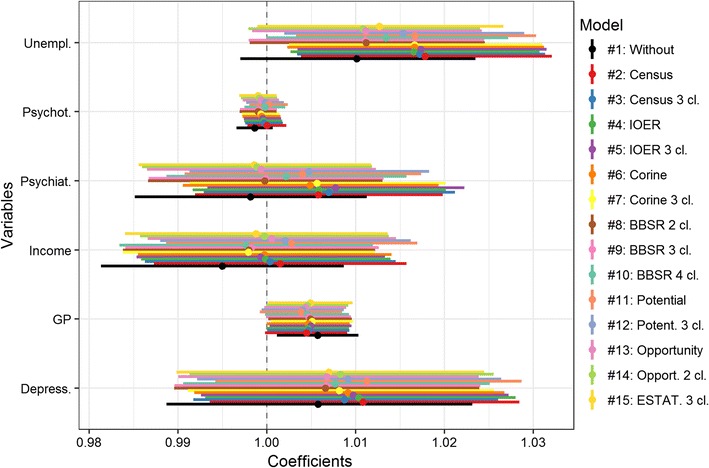
Relative risk of the covariates including the 95% CI across the models

The effects of the covariates are presented in Fig. 4. The models indicate differences in the support and effect size of the covariates. Focusing on the best performing model (i.e., model 11) (Additional file 1: Table A3), the associations are as follows: Unemployment rate is positively associated (1.017, 95% CI 1.003; 1.030), whereas income and depression prevalence appear not related. In contrast to psychiatrists and psychotherapists, who are also not supported through the model, the number of GPs per 100,000 persons has a positive but weak association with suicide risk (1.005, 95% CI 1.000; 1.009).

**Fig. 5:**
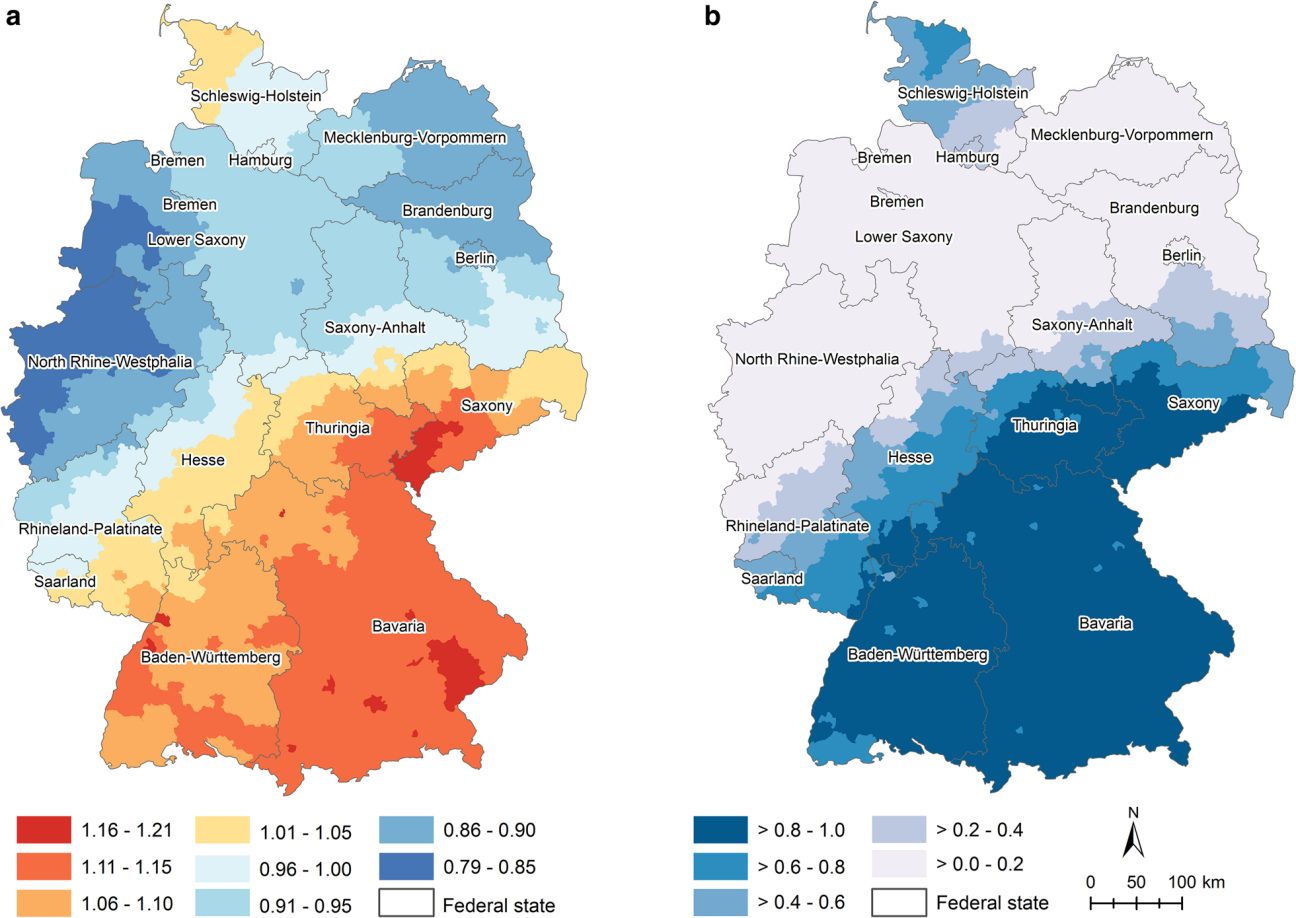
Residual relative risks per district (**a**) and the posterior probability (**b**). Both maps refer to model 11

The residual relative risk not explained by the covariates and the corresponding posterior probability are shown in Fig. 5. Striking patterns following a north–south trend are observable. Compared to the German-wide risk, districts located in the south-eastern parts (e.g., Bavaria) show the highest suicide risk compared to more central areas (e.g., North Rhine–Westphalia).

## Discussion

### Principle findings

This study rigorously examined the extent to which different urbanicity indicators affect urban–rural suicide associations. Our analyses showed that urban–rural indicators are significantly positively associated. For example, the correlation between the population potential score and population density is 0.902, which is higher than the 0.620 for England and Wales [21]. These differences may result from the application of varying distance metrics and/or distance decay parameters required for the population potential score. In contrast to Euclidean distances [21], which are known to underestimate actual street distances [61], we utilized the more accurate street network distances.

As our regressions confirmed, there is sound evidence that the residents of German rural areas face a higher suicide risk than those in urbanized areas [9]. The observed urban–rural divide in suicide is consistent with other studies [7, 24, 45]. In Portugal, for instance, rurality is positively correlated with suicide mortality [38]. However, in Belgium urbanicity was not significantly associated with lower suicide risk, whereas Canadian cities seem to face an elevated risk [62]. A similar reverse effect of pronounced suicides in urbanized areas was found in Danish register analyses [29], ecological studies in England and Wales [21], and among US adults [19]. These contradictory findings might be caused by inconsistent definitions of “urbanicity” [23].

Different operationalizations of urbanicity influenced the size of the urban–rural effect on suicide mortality and/or eliminated its significance, but not the effect sign. To circumvent arbitrary class breaks, we applied a clustering approach. A low number of regions is consistently preferred, which is in contrast to other studies [19, 22]. In keeping with others [3], dichotomous representations in urban and rural areas across Germany seem less suitable, although widespread. It stands out that continuous urban–rural indicators perform better than those on an ordinal scale [3]. Rural areas with densification tendencies are not found to have suicide rates different from those in the countryside. Only extremes of urbanicity levels indicate clear differences.

Our results imply that regional planning-based urban–rural taxonomies (i.e., BBSR and ESTAT) do not capture suicide disparities appropriately. The model performance is inferior even to that of the model without an urban–rural indicator. Planning regions are not designed to reflect health inequalities and they potentially mask internal heterogeneities [31, 32]. Thus, the application of planning regions raises practical concerns regarding the modifiable area unit problem, in that variation in zoning and/or spatial scales affects suicide–urbanicity relations [63]. Measures based on built-up areas and transportation infrastructure, consistently lead to significant urban–rural inequalities in suicide. The better model performance of the measure using the precise ATKIS data, rather than the Corine data, suggests that precise input data should be considered, even though the output measure is aggregated at a district level. However, the slightly different timestamps of the indicators (2011 vs. 2012) might contribute to the mismatch in the results. Whereas ATKIS data are available only for Germany, Corine data [39], even though they are limited by a spatial resolution of 100 m and a minimum mapping unit of 25 hectares [64], seem useful for transnational European research because they assure consistent indicators.

Our results show that modeling urban–rural differences in suicide mortality by means of population density is the second best choice, thus legitimating its widespread application [9, 11, 12, 14, 37, 38]. An advantage of population-based indicators is that the data are easily accessible, annually updated, and available in most countries, which facilitates inter-country comparability between studies. However, population density is a place-based representation and does not consider interaction with other areas. With only average model fits, we could not find evidence that the cumulative population opportunity index (i.e., the number of people within a 60-min drive) should be preferred to population density. As this indicator considers a frequently used 60-min travel time [36], it may be that that this threshold value is less suitable for densely populated Germany. In contrast, the population potential score is more realistic, as the nearby population is weighted more heavily than the population living farther away [35], which could explain the highest gain in model fit [21]. However, this improved fit is at the expense of a less straightforward interpretation (e.g., due to distance decay effects).

### Strengths and limitations

This study broke new ground, and several of its strengths need to be emphasized. It was the first study to systematically address the influence of 14 different urban–rural indicators on suicide mortality. Second, it contributes to the limited number of ecological studies carried out in continental Europe. To the best of our knowledge, we pioneered research on urban–rural inequalities in suicide mortality in Germany. Third, besides a comprehensive set of covariates, we controlled for depression prevalence [11], in contrast to most other area-based studies [7, 12]. Fourth, due to the sample size, our statistical results are deemed to be robust. Fifth, we utilized the latest advances in statistical analyses [54] and our models successfully integrate spatial autocorrelation [55].

Several limitations should be taken into account when interpreting the results. First, since the data were pooled over time, it was not possible to examine growing or shrinking urban–rural disparities [19], which would require the application of space–time models [9]. Second, when dealing with nationwide studies, the influence of risk and protective factors is likely to vary spatially [65]. Third, because the data used in this research are based on areal units (i.e., districts), the modifiable areal unit problem might have influenced the results [63] and inference at the individual level may not be valid because of ecological fallacy [66]. Fourth, although suicide in high-income countries is more prevalent in elderly males [10], data protection issues prevented a stratification by age and gender, and thus the calculation of age-adjusted mortality rates [22, 38]. However, the impact of standardization on outcomes in geographical correlational studies seems to be minor [67]. Fifth, congruent with the majority of studies [7, 37, 46], we assumed that people are only exposed to the actual place of residence (i.e., their district). As suicide develops over the lifetime, future studies should be longitudinal and put central people’s residential history over their life course. Finally, our results obtained for Germany may not be generalizable to other countries, and verification merits further research.

## Conclusion

Germany faces urban–rural inequalities in suicide mortality. We found that rurality is related to higher suicide risk. This association is consistent across several urban–rural indicators. We also found evidence that the selected indicator determines whether or not inequalities in suicide mortality are demonstrated. Both the effect size and the statistical significance varied across different urbanicity operationalizations, but the direction of the estimated urban–rural effect remained unaffected. Continuous indicators along the urban–rural continuum are auspicious, supporting the notion that urban areas continuously transit into rural ones. For future replication in other studies, the findings suggest that accessibility indicators, such as the population potential, perform best and that population density also performs well. Dichotomous and ordinally scaled urban–rural indicators are of limited value. The majority of such urban–rural taxonomies have failed to show significant differences in suicide risk while pointing to low model fits. We encourage researchers to go beyond a single representation of urbanicity/rurality when exploring suicide inequalities spatially, and to pay attention to how diverse urban–rural indicators may alter model outputs. Further, we recommend using sensitivity analyses to investigate whether results are consistent across urban–rural indicators.

## Additional file

**Additional file 1: Table A1.** Descriptive statistics. **Table A2.** Correlation between urbanicity indicators. **Table A3.** Regression results of the best performing model 11. **Figure A1.** Model performances for different urban–rural measures.

## Abbreviations

ATKIS: Amtliche Topographisch-Kartographische Informations System
CPO: conditional predictive ordinate
CI: credibility intervals
DIC: deviance information criterion
IOER: Leibniz Institute of Ecological Urban and Regional Development

## Footnotes

1 Based on remote sensing images, land-use was classified into 44 classes at a spatial resolution of 100 × 100 m (Copernicus, 2016). The indicator preparation considers the following classes: 111 (continuous urban fabric), 112 (discontinuous urban fabric), 121 (industrial and commercial units), 122 (road, rail networks, and associated land), 123 (ports), and 124 (airports).

2 The cumulative opportunity index CO for area *i* refers to the number of people *P* in reach along the street network between *i* and all opportunities *j* within a relevant threshold car-based driving time *t* (i.e., 60 min): *CO*_*i*_ = Σ_*j*_ *P*_*j*_ where *t*_*ij*_ ≤ *t*. Higher *CO*_*i*_ scores refer to a better accessibility.

3 In contrast to the cumulative opportunity index, the potential measure *P*_*i*_ does not take any threshold distance into account but considers all opportunities *j* in combination with a distance decay effect (i.e., the interaction declines with increasing distance or travel time). *P*_*i*_ = Σ_*j*_ *P*_*j*_ *F*(*t*_*ij*_) where, *P*_*j*_ refers to the number of people and *F*(*t*_*ij*_) represents car-based driving time to the negative power of 2.

## References

[1] Cyril S, Oldroyd JC, Renzaho A (2013). Urbanisation, urbanicity, and health: a systematic review of the reliability and validity of urbanicity scales. BMC Public Health BioMed Cent.

[2] Heinz A, Deserno L, Reininghaus U (2013). Urbanicity, social adversity and psychosis. World Psychiatry.

[3] Peen J, Schoevers RA, Beekman AT, Dekker J (2010). The current status of urban–rural differences in psychiatric disorders. Acta Psychiatr Scand.

[4] World Health Organization (2014). Preventing suicide: a global imperative.

[5] Hirsch JK, Cukrowicz KC (2014). Suicide in rural areas: an updated review of the literature. J Rural Ment Heal.

[6] Hong J, Knapp M (2013). Geographical inequalities in suicide rates and area deprivation in South Korea. J Ment Health Policy Econ.

[7] Hsu C-Y, Chang S-S, Lee EST, Yip PSF (2015). Geography of suicide in Hong Kong: spatial patterning, and socioeconomic correlates and inequalities. Soc Sci Med.

[8] Searles VB, Valley MA, Hedegaard H, Betz ME (2014). Suicides in urban and rural counties in the United States, 2006–2008. Crisis.

[9] Helbich M, Plener P, Hartung S, Blüml V (2017). Spatiotemporal suicide risk in Germany: a longitudinal study 2007–2011. Sci Rep.

[10] Turecki G, Brent DA (2016). Suicide and suicidal behaviour. Lancet.

[11] Blüml V, Helbich M, Mayr M, Turnwald R, Vyssoki B, Lewitzka U (2017). Antidepressant sales and regional variations of suicide mortality in Germany. J Psychiatr Res.

[12] Chan CH, Caine ED, You S, Yip PSF (2015). Changes in South Korean urbanicity and suicide rates, 1992 to 2012. BMJ Open.

[13] Helbich M, Leitner M, Kapusta ND (2015). Lithium in drinking water and suicide mortality: interplay with lithium prescriptions. Br J Psychiatry.

[14] Kapusta ND, Zorman A, Etzersdorfer E, Ponocny-Seliger E, Jandl-Jager E, Sonneck G (2008). Rural–urban differences in Austrian suicides. Soc Psychiatry Psychiatr Epidemiol.

[15] Hawton K, Casañas Comabella C, Haw C, Saunders K (2013). Risk factors for suicide in individuals with depression: a systematic review. J Affect Disord.

[16] Haw C, Hawton K, Niedzwiedz C, Platt S (2013). Suicide clusters: a review of risk factors and mechanisms. Suicide Life Threat Behav.

[17] Rehkopf DH, Buka SL (2006). The association between suicide and the socio-economic characteristics of geographical areas: a systematic review. Psychol Med.

[18] Lambert KG, Nelson RJ, Jovanovic T, Cerdá M (2015). Brains in the city: neurobiological effects of urbanization. Neurosci Biobehav Rev.

[19] Fontanella CA, Hiance-Steelesmith DL, Phillips GS, Bridge JA, Lester N, Sweeney HA (2015). Widening rural–urban disparities in youth suicides, United States, 1996–2010. JAMA Pediatr.

[20] Judd F, Cooper A-M, Fraser C, Davis J (2006). Rural suicide—people or place effects?. Aust NZ J Psychiatry.

[21] Middleton N, Gunnell D, Frankel S, Whitley E, Dorling D (2003). Urban–rural differences in suicide trends in young adults: England and Wales, 1981–1998. Soc Sci Med.

[22] Page A, Morrell S, Taylor R, Dudley M, Carter G (2007). Further increases in rural suicide in young Australian adults: Secular trends, 1979–2003. Soc Sci Med.

[23] Pearce J, Barnett R, Jones I (2007). Have urban/rural inequalities in suicide in New Zealand grown during the period 1980–2001?. Soc Sci Med.

[24] Phillips JA (2013). Factors associated with temporal and spatial patterns in suicide rates across US states, 1976–2000. Demography.

[25] Singh GK, Siahpush M (2002). Increasing rural–urban gradients in US suicide mortality, 1970–1997. Am J Public Health.

[26] Yip PSF, Callanan C, Yuen HP (2000). Urban/rural and gender differentials in suicide rates: East and West. J Affect Disord.

[27] Kim M-H, Jung-Choi K, Jun H-J, Kawachi I (2010). Socioeconomic inequalities in suicidal ideation, parasuicides, and completed suicides in South Korea. Soc Sci Med.

[28] Kapusta ND, Posch M, Niederkrotenthaler T, Fischer-Kern M, Etzersdorfer E, Sonneck G (2010). Availability of mental health service providers and suicide rates in Austria: a nationwide study. Psychiatr Serv.

[29] Mortensen PB, Agerbo E, Erikson T, Qin P, Westergaard-Nielsen N (2000). Psychiatric illness and risk factors for suicide in Denmark. Lancet.

[30] Goodall CR, Kafadar K, Tukey JW (1998). Competing and using moral versus urban measures in statistical applications. Am Stat.

[31] Hall SA, Kaufman JS, Ricketts TC (2006). Defining urban and rural areas in US epidemiologic studies. J Urban Heal.

[32] Hart LG, Larson EH, Lishner DM (2005). Rural definitions for health policy and research. Am J Public Health.

[33] Perlin R (2010). Theoretical approaches of methods to delimitate rural and urban areas. Eur Countrys.

[34] Pászto V, Brychtová A, Tuček P, Marek L, Burian J (2015). Using a fuzzy inference system to delimit rural and urban municipalities in the Czech republic in 2010. J Maps.

[35] Handy SL, Niemeier DA (1997). Measuring accessibility: an exploration of issues and alternatives. Environ Plan A.

[36] Geurs KT, van Eck JR. Accessibility measures: review and applications. Rijksinstituut voor Volksgezondheid en Milieu RIVM; 2001.

[37] Chang S-S, Sterne JAC, Wheeler BW, Lu T-H, Lin J-J, Gunnell D (2011). Geography of suicide in Taiwan: spatial patterning and socioeconomic correlates. Health Place.

[38] Santana P, Costa C, Cardoso G, Loureiro A, Ferrão J (2015). Suicide in Portugal: spatial determinants in a context of economic crisis. Health Place.

[39] Feranec J, Jaffrain G, Soukup T, Hazeu G (2010). Determining changes and flows in European landscapes 1990–2000 using CORINE land cover data. Appl Geogr.

[40] Chen J, Cao X, Peng S, Ren H (2017). Analysis and applications of GlobeLand30: a review. ISPRS Int J Geo-Inf.

[41] Mitchell R, Astell-Burt T, Richardson EA. A comparison of green space indicators for epidemiological research. J Epidemiol Community Health 2011;jech–2010. 10.1136/jech.2010.119172 21296907

[42] Hegerl U, Mergl R, Doganay G, Reschke K, Rummel-Kluge C (2013). Why has the continuous decline in German suicide rates stopped in 2007?. PLoS One.

[43] Etzersdorfer E, Bronisch T (2004). Der wissenschaftliche Beirat des nationalen Suizidpräventionsprogrammes. Suizidprophylaxe.

[44] O’Farrell IB, Corcoran P, Perry IJ (2016). The area level association between suicide, deprivation, social fragmentation and population density in the Republic of Ireland: a national study. Soc Psychiatry Psychiatr Epidemiol.

[45] Cheung YTD, Spittal MJ, Pirkis J, Yip PSF (2012). Spatial analysis of suicide mortality in Australia: investigation of metropolitan-rural-remote differentials of suicide risk across states/territories. Soc Sci Med.

[46] Pirkola S, Sund R, Sailas E, Wahlbeck K (2009). Community mental-health services and suicide rate in Finland: a nationwide small-area analysis. Lancet.

[47] Statistisches Bundesamt. Todesursachenstatistik. Todesursachen in Deutschland [Internet]. Wiesbaden; 2017. http://www.destatis.de/DE/Publikationen/Qualitaetsberichte/Gesundheitswesen/Todesursachen.pdf?__blob=publicationFile.

[48] Guagliardo MF (2004). Spatial accessibility of primary care: concepts, methods and challenges. Int J Health Geogr.

[49] Eurostat. Urban–rural typology [Internet]. 2017. http://ec.europa.eu/eurostat/statistics-explained/index.php/Urban-rural_typology.

[50] Gallego FJ (2010). A population density grid of the European Union. Popul Environ.

[51] Helbich M, Blüml V, Leitner M, Kapusta ND (2013). Does altitude moderate the impact of lithium on suicide? A spatial analysis of Austria. Geospat Health.

[52] Jain AK (2010). Data clustering: 50 years beyond K-means. Pattern Recognit Lett.

[53] Spiegelhalter DJ, Best NG, Carlin BP, Linde A (2014). The deviance information criterion: 12 years on. J R Stat Soc Ser B.

[54] Rue H, Martino S, Chopin N (2009). Approximate bayesian inference for latent Gaussian models by using integrated nested Laplace approximations. J R Stat Soc Ser B.

[55] Lawson AB (2013). Bayesian disease mapping: hierarchical modeling in spatial epidemiology.

[56] Wakefield J (2006). Disease mapping and spatial regression with count data. Biostatistics.

[57] Besag J, York J, Mollié A (1991). Bayesian image restoration, with two applications in spatial statistics. Ann Inst Stat Math.

[58] Earnest A, Morgan G, Mengersen K, Ryan L, Summerhayes R, Beard J (2007). Evaluating the effect of neighbourhood weight matrices on smoothing properties of conditional autoregressive (CAR) models. Int J Health Geogr BioMed Cent.

[59] Richardson S, Thomson A, Best N, Elliott P (2004). Interpreting posterior relative risk estimates in disease-mapping studies. Environ Health Perspect.

[60] Rue H, Riebler A, Sørbye SH, Illian JB, Simpson DP, Lindgren FK (2017). Bayesian computing with INLA: a review. Annu Rev Stat Its Appl Annu Rev.

[61] Stigell E, Schantz P (2011). Methods for determining route distances in active commuting—their validity and reproducibility. J Transp Geogr.

[62] Rhee Y, Houttekier D, MacLeod R, Wilson DM, Cardenas-Turanzas M, Loucka M (2016). International comparison of death place for suicide. A population-level eight country death certificate study. Soc Psychiatry Psychiatr Epidemiol.

[63] Schuurman N, Bell N, Dunn JR, Oliver L (2007). Deprivation indices, population health and geography: an evaluation of the spatial effectiveness of indices at multiple scales. J Urban Heal.

[64] Copernicus. Corine land cover 2012 [Internet]. 2016. http://land.copernicus.eu/pan-european/corine-land-cover/clc-2012.

[65] Helbich M, Leitner M, Kapusta ND (2012). Geospatial examination of lithium in drinking water and suicide mortality. Int J Health Geogr.

[66] McLaren L, Hawe P (2005). Ecological perspectives in health research. J Epidemiol Community Heal.

[67] Kapusta ND, Mossaheb N, Etzersdorfer E, Hlavin G, Thau K, Willeit M (2011). Lithium in drinking water and suicide mortality. Br J Psychiatry.

