# Supplementary material for: Urban–rural inequalities in suicide mortality: a comparison of urbanicity indicators
Source: Int J Health Geogr. 2017 Oct 30;16:39. doi: 10.1186/s12942-017-0112-x (PMC5663034; doi:10.1186/s12942-017-0112-x)
Supplement: Supplementary file 1 — Additional file 1: Table A1. Descriptive statistics. Table A2. Correlation between urbanicity indicators. Table A3. Regression results of the best performing model 11. Figure A1. Model performances for different urban–rural measures. [file 12942_2017_112_MOESM1_ESM.docx]

**Additional file 1**

**Urban–Rural Inequalities in Suicide Mortality: A Comparison of Urbanicity Indicators**

Marco Helbich, Victor Blüml, Tom de Jong, Paul L Plener, Mei-Po Kwan, Nestor D Kapusta

Table A1: Descriptive statistics.

| *Numeric variables* | Minimum | 25% quartile | Median | 75% quartile | Maximum. | Standard deviation |
| --- | --- | --- | --- | --- | --- | --- |
| Suicides (average 2007–11) | 4.0 | 13.0 | 19.0 | 27.0 | 358.0 | 26.2 |
| Population (average 2007–11, in 1,000 persons) | 34.3 | 105.2 | 148.5 | 238.0 | 3,415.0 | 229.9 |
| Disposable income 2011 (in €1,000) | 14.8 | 18.0 | 19.8 | 21.2 | 33.0 | 2.5 |
| Unemployment rate 2011 (in %) | 1.4 | 4.0 | 5.9 | 8.5 | 17.0 | 3.2 |
| Depression prevalence 2011 (in %) | 7.6 | 10.6 | 11.7 | 12.8 | 20.0 | 1.8 |
| GPs 2011 (per 100,000 persons) | 46.1 | 59.3 | 64.0 | 68.2 | 97.0 | 7.6 |
| Psychiatrists 2011 (per 100,000 persons) | 0.0 | 3.4 | 4.3 | 6.0 | 19.0 | 3.1 |
| Psychotherapists 2011 (per 100,000 persons) | 1.7 | 10.9 | 16.1 | 24.8 | 130.0 | 17.1 |
| Census 2011 (population density) | 37.1 | 113.0 | 196.2 | 637.8 | 4,262.0 | 657.6 |
| IOER 2011 (built-up areas, in %) | 4.0 | 8.2 | 10.9 | 22.4 | 63.0 | 12.9 |
| Corine 2012 (built-up areas, in %) | 2.4 | 5.8 | 8.6 | 22.2 | 72.0 | 14.6 |
| Population potential scores 2011 | 0.54 | 1.3 | 1.8 | 3.2 | 14.0 | 2.2 |
| Cumulative opportunity index 2011 (in 1,000 persons) | 190 | 1,683 | 2,721 | 4,480 | 14,269 | 3,109 |
| *Categorical variables* | Frequency (%) |  |  |  |  |  |
| Census 2011 (population density clustered in 3 regions) | 1: 189 (47.0) | 2: 111 (27.6) | 3: 102 (25.4) |  |  |  |
| IOER 2011 (built-up areas clustered in 3 regions) | 1: 172 (42.8) | 2: 13 (30.6) | 3: 107 (26.6) |  |  |  |
| Corine 2012 (built-up areas clustered in 3 regions) | 1: 171 (42.5) | 2: 118 (29.4) | 3: 113 (28.1) |  |  |  |
| BBSR 2011 (typology with 2 regions) | 1: 199 (49.5) | 2: 203 (50.5) |  |  |  |  |
| BBSR 2011 (typology with 3 regions) | 1: 96 (23.9) | 2: 103 (25.6) | 3: 203 (50.5) |  |  |  |
| BBSR 2011 (typology with 4 regions) | 1: 96 (23.9) | 2: 103 (25.6) | 3: 137 (34.1) | 4: 66 (16.4) |  |  |
| Population potential scores 2011 (clustered in 3 regions) | 1: 184 (45.8) | 2: 141 (35.1) | 3: 77 (19.2) |  |  |  |
| Cumulative opportunity index 2011 (clustered in 2 regions) | 1: 216 (53.7) | 2: 186 (46.3) |  |  |  |  |
| ESTAT 2011 (typology with 3 regions) | 1: 113 (28.1) | 2: 200 (49.8) | 3: 89 (22.1) |  |  |  |

Table A2: Correlation between urbanicity indicators.

|  | #2: Census (logged pop. density) | #3: Census (pop. density clustered in 3 regions) | #4: IOER (logged built-up areas %) | #5: IOER (built-up areas clustered in 3 regions) | #6: Corine (logged built-up areas %) | #7: Corine (built-up areas clustered in 3 regions) | #8: BBSR (typol. with 2 regions) | #9: BBSR (typol. with 3 regions) | #10: BBSR (typol. with 4 regions) | #11: Pop. potential scores (logged) | #12: Pop. potential scores (clustered in 3 regions) | #13: Cumulative opport. index (logged) | #14: Cumulative opport. index (clustered in 2 regions) | #15: ESTAT (typol. with 3 regions) |
| --- | --- | --- | --- | --- | --- | --- | --- | --- | --- | --- | --- | --- | --- | --- |
| #2: Census (logged pop. density) |  | 0.927 | 0.967 | 0.907 | 0.956 | 0.902 | 0.708 | 0.743 | 0.814 | 0.902 | 0.868 | 0.605 | 0.507 | 0.687 |
| #3: Census (pop. density clustered in 3 regions) | <0.001 |  | 0.902 | 0.903 | 0.899 | 0.893 | 0.700 | 0.678 | 0.749 | 0.858 | 0.849 | 0.537 | 0.470 | 0.620 |
| #4: IOER (logged built-up areas %) | <0.001 | <0.001 |  | 0.935 | 0.966 | 0.915 | 0.656 | 0.686 | 0.762 | 0.869 | 0.840 | 0.568 | 0.469 | 0.647 |
| #5: IOER (built-up areas clustered in 3 regions) | <0.001 | <0.001 | <0.001 |  | 0.914 | 0.928 | 0.641 | 0.642 | 0.718 | 0.827 | 0.804 | 0.510 | 0.410 | 0.612 |
| #6: Corine (logged built-up areas %) | <0.001 | <0.001 | <0.001 | <0.001 |  | 0.936 | 0.647 | 0.672 | 0.750 | 0.878 | 0.843 | 0.569 | 0.477 | 0.669 |
| #7: Corine (built-up areas clustered in 3 regions) | <0.001 | <0.001 | <0.001 | <0.001 | <0.001 |  | 0.606 | 0.615 | 0.696 | 0.821 | 0.795 | 0.494 | 0.403 | 0.617 |
| #8: BBSR (typol. with 2 regions) | <0.001 | <0.001 | <0.001 | <0.001 | <0.001 | <0.001 |  | 0.944 | 0.900 | 0.669 | 0.657 | 0.568 | 0.500 | 0.628 |
| #9: BBSR (typol. with 3 regions) | <0.001 | <0.001 | <0.001 | <0.001 | <0.001 | <0.001 | <0.001 |  | 0.953 | 0.685 | 0.655 | 0.592 | 0.524 | 0.645 |
| #10: BBSR (typol. with 4 regions) | <0.001 | <0.001 | <0.001 | <0.001 | <0.001 | <0.001 | <0.001 | <0.001 |  | 0.755 | 0.721 | 0.603 | 0.528 | 0.664 |
| #11: Pop. potential scores (logged) | <0.001 | <0.001 | <0.001 | <0.001 | <0.001 | <0.001 | <0.001 | <0.001 | <0.001 |  | 0.924 | 0.775 | 0.639 | 0.663 |
| #12: Pop. potential scores (clustered in 3 regions) | <0.001 | <0.001 | <0.001 | <0.001 | <0.001 | <0.001 | <0.001 | <0.001 | <0.001 | <0.001 |  | 0.703 | 0.629 | 0.650 |
| #13: Cumulative opport. index (logged) | <0.001 | <0.001 | <0.001 | <0.001 | <0.001 | <0.001 | <0.001 | <0.001 | <0.001 | <0.001 | <0.001 |  | 0.864 | 0.584 |
| #14: Cumulative opport. index (clustered in 2 regions) | <0.001 | <0.001 | <0.001 | <0.001 | <0.001 | <0.001 | <0.001 | <0.001 | <0.001 | <0.001 | <0.001 | <0.001 |  | 0.507 |
| #15: ESTAT (typol. with 3 regions) | <0.001 | <0.001 | <0.001 | <0.001 | <0.001 | <0.001 | <0.001 | <0.001 | <0.001 | <0.001 | <0.001 | <0.001 | <0.001 |  |

*p*-values are given in the lower diagonal.

Table A3: Regression results of the best performing model 11.

| *Numeric variables* | Relative  risk | 2.5%  CI | 97.5%  CI |
| --- | --- | --- | --- |
| Intercept | 0.628* | 0.406 | 0.972 |
| Disposable income (in €1,000) | 1.003 | 0.989 | 1.017 |
| Unemployment rate (in %) | 1.017* | 1.003 | 1.030 |
| Depression prevalence (in %) | 1.011 | 0.994 | 1.029 |
| GPs (per 100,000 persons) | 1.004* | 1.000 | 1.009 |
| Psychiatrists (per 100,000 persons) | 1.004 | 0.991 | 1.017 |
| Psychotherapists (per 100,000 persons) | 1.000 | 0.998 | 1.002 |
| Population potential score | 0.903* | 0.854 | 0.955 |

“*” refers to a strong statistical evidence


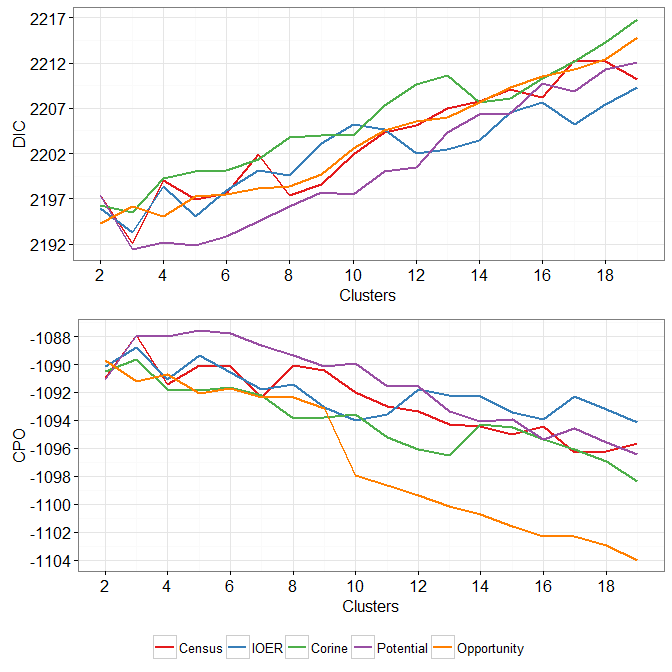


Figure A1: Model performances for different urban–rural measures.
